# Association of Race With Pulse Oximetry Accuracy in Hospitalized Children

**DOI:** 10.1001/jamanetworkopen.2022.4584

**Published:** 2022-03-31

**Authors:** Erica Andrist, Mark Nuppnau, Ryan P. Barbaro, Thomas S. Valley, Michael W. Sjoding

**Affiliations:** 1 Division of Pediatric Critical Care Medicine, C.S. Mott Children’s Hospital, Ann Arbor, Michigan; 2 Division of Pulmonary and Critical Care Medicine, Department of Internal Medicine, Ann Arbor, Michigan

## Abstract

This cross-sectional study assesses differences in the accuracy of oxygen saturation measured by pulse oximetry among Black and White pediatric patients.

## Introduction

Oxygen saturation measured by pulse oximetry (SpO_2_) is used for triage and management of acute illness in hospitalized children. Adult data indicate that pulse oximetry does not detect hypoxemia as frequently in Black patients as in White patients.^1,2^ We aimed to investigate the frequency of occult hypoxemia in children and assess variability by patient race.

## Methods

This cross-sectional study conducted at a single center in Ann Arbor, Michigan, analyzed patients aged 17 years or younger admitted between January 1, 2015, and December 31, 2020, who self-identified or were identified by a parent as Black or White. We extracted patient race, age, SpO_2_, arterial blood gas (ABG) data, respiratory support, vasoactive support, and diagnosis information from electronic health records. The University of Michigan Medical School Institutional Review Board exempted the study from approval and informed consent because it constituted secondary research. This study followed the STROBE reporting guideline.

We compared SpO_2_ readings with arterial oxygen saturation (SaO_2_) readings directly measured by ABG if the ABG measurement was performed within 10 minutes at the same respiratory support. Exclusion criteria are presented in the eTable in the Supplement. We used the Vasoactive Infusion Score^3^ to adjust for cardiovascular severity of illness.

We evaluated the mean pulse oximeter bias (SpO_2_ – SaO_2_) and frequency of occult hypoxemia (SpO_2_ ≥92% and SaO_2_ <88%) by patient race. Statistical analyses were conducted using Stata, version 16 (StataCorp LLC). Significance was set at 2-sided *P* < .05 (eMethods and eAppendix in the Supplement).

## Results

We analyzed 9023 SpO_2_-SaO_2_ pairs from 1061 total patients (878 [82.8%] White; 183 [17.2%] Black; 572 [53.9%] male; mean [SD] age, 7 [6] years) (Table). The median time between SpO_2_ and SaO_2_ readings was 4 minutes (range, 0 to 9 minutes) for both groups. The overall mean (SD) pulse oximeter bias was 3.5% (5.0%) among White patients and 4.3% (5.0%) among Black patients (*P* < .001). The frequency of occult hypoxemia was 5.8% (95% CI, 4.6%-7.3%) among White patients and 9.6% (95% CI, 6.3%-14.5%) among Black patients (Figure). After adjusting for age, sex, SpO_2_, and Vasoactive Infusion Score, the odds ratio for occult hypoxemia among SpO_2_ measurements for Black patients was 2.16 (95% CI, 1.36-3.44) compared with White patients. At the patient level, 134 of 860 White patients (15.6%; 95% CI, 13.3%-18.2%) and 38 of 180 Black patients (21.1%; 95% CI, 15.7%-27.7%) had occult hypoxemia episodes. After adjusting for the same covariates, the odds ratio for occult hypoxemia was 1.79 (95% CI, 1.07-3.02) for Black patients compared with White patients.

**Table.** Characteristics of the Study Sample by Race

Characteristic	Hospitalizations, No. (%)^a^			*P* value^b^
	White	Black	Total	
Hospitalizations^c^	934 (83.4)	186 (16.6)	1120 (100)	NA
Unique patients	878 (82.8)	183 (17.2)	1061 (100)	NA
Sex				
Female	426 (45.6)	90 (48.4)	516 (46.1)	.54
Male	508 (54.4)	96 (51.6)	604 (53.9)	
Patient age				
<6 mo	152 (16.3)	33 (17.7)	185 (16.5)	.98
6 to <12 mo	83 (8.9)	16 (8.6)	99 (8.8)	
12 to <24 mo	75 (8.0)	12 (6.5)	87 (7.8)	
2 to <5 y	152 (16.3)	29 (15.6)	181 (16.2)	
5 to <12 y	207 (22.2)	42 (22.6)	249 (22.2)	
12 to 17 y	265 (28.4)	54 (29.0)	319 (28.5)	
Maximum respiratory support				
None	35 (3.7)	8 (4.3)	43 (3.8)	.002
Supplemental oxygen	313 (33.5)	36 (19.4)	349 (31.2)	
Noninvasive positive pressure	43 (4.6)	11 (5.9)	54 (4.8)	
Invasive mechanical ventilation	543 (58.1)	131 (70.4)	674 (60.2)	
Maximum VIS				
0-5	551 (59.0)	115 (61.8)	666 (59.5)	.51
6-10	120 (12.8)	18 (9.7)	138 (12.3)	
11-20	111 (11.9)	26 (14.0)	137 (12.2)	
>20	152 (16.3)	27 (14.5)	179 (16.0)	
SpO_2_-SaO_2_ pairs, No./total No. (%)^d^	7018/9023 (77.8)	2005/9023 (22.2)	9023/9023 (100)	NA
SpO_2_ values, No./total No. (%)				
<88%	626/7018 (8.9)	248/2005 (12.4)	874/9023 (9.7)	.71
≥88%	6392/7018 (91.1)	1757/2005 (87.6)	8149/9023 (90.3)	
SpO_2_ – SaO_2_, mean (SD)	3.5 (5.0)	4.3 (5.0)	3.7 (5.0)	<.001
SaO_2_ <88% and SpO_2_ ≥88%, No./total No. (%)				
SpO_2_ of 88%-91%	188/334 (56.3)	74/106 (69.8)	262/440 (59.6)	NA
SpO_2_ of 92%-96%	248/1845 (13.4)	127/433 (29.3)	375/2278 (16.5)	
SpO_2_ >96%	104/4213 (2.5)	32/1218 (2.6)	136/5431 (2.5)	
Occult hypoxemia, No./total No. (%)^e^	352/6058 (5.8)	159/1651 (9.6)	511/7709 (6.6)	.03

Abbreviations: NA, not applicable; SaO_2_, arterial oxygen saturation; SpO_2_, oxygen saturation measured by pulse oximetry; VIS, Vasoactive Infusion Score.

^a^ Some percentages may not sum to 100% owing to rounding.

^b^ *P* values for the patient-level analysis were calculated using the χ^2^ test or analysis of variance; *P* values for the SpO_2_ measurement-level analysis were calculated using mixed models to adjust for clustering within patients.

^c^ Sex, age, maximum respiratory support, and VIS are reported for each hospitalization.

^d^ The sample included SpO_2_ and SaO_2_ directly measured by arterial blood gas analysis. Pairs were measured within 10 minutes at the same level of respiratory support.

^e^ Occult hypoxemia was defined as SaO_2_ less than 88% in the presence of SpO_2_ of 92% or greater.

**Figure.**
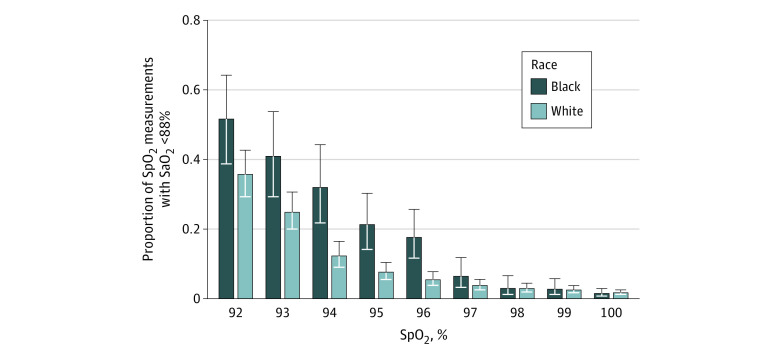
Racial Differences in the Proportion of Oxygen Saturation Measured by Pulse Oximetry (SpO_2_)–Arterial Oxygen (SaO_2_) Pairs Demonstrating Occult Hypoxemia Vertical lines indicate 95% CIs.

## Discussion

In this study, 21.1% of Black children experienced arterial hypoxemia despite a normal SpO_2_ reading; occult hypoxemia occurred more frequently than in White children. Among SpO_2_-Sao_2_ pairs, the odds of the SpO_2_ reading failing to detect arterial hypoxemia were more than twice as great if the pair had come from a Black child. Underrecognition and undertreatment of hypoxemia in Black children may contribute to racial disparities in respiratory illness outcomes.^4,5,6^ We observed higher mean pulse oximeter bias among all children than has been reported in adults.^2^

This study has limitations. Because pediatric ABG values are rarely obtained outside critical care settings, all study patients were likely critically ill. Clinically significant changes could have occurred in the 10-minute window between obtaining SpO_2_ and SaO_2_ values. Black children required more respiratory support than White children, potentially confounding the results, although the findings were not different in adjusted analysis. In addition, self-reported race encompasses many phenotypes. Future research may explore more nuanced associations between pulse oximetry accuracy and skin pigmentation and whether pulse oximeter bias is associated with outcomes. Further evaluation of racial differences in pediatric pulse oximetry accuracy in children and investigation of potential clinical outcomes are warranted.

## Notes

Supplement. **eMethods.** Exclusion Criteria **eTable.** Grouping of *International Classification of Disease, Version 10* Codes Used to Exclude Patients With Ductal-Dependent Cardiac Lesions From Analysis **eAppendix.** Stata Log Files
