# Supplementary material for: Association of Race With Pulse Oximetry Accuracy in Hospitalized Children
Source: JAMA Netw Open. 2022 Mar 31;5(3):e224584. doi: 10.1001/jamanetworkopen.2022.4584 (PMC8972025; doi:10.1001/jamanetworkopen.2022.4584)
Supplement: Supplement. — eMethods. Exclusion Criteria eTable. Grouping of International Classification of Disease, Version 10 Codes Used to Exclude Patients With Ductal-Dependent Cardiac Lesions From Analysis eAppendix. Stata Log Files [file jamanetwopen-e224584-s001.pdf]

Supplementary Online Content

Andrist E, Nuppnau M, Barbaro RP, Valley TS, Sjoding MW. Association of race with pulse oximetry accuracy in hospitalized children. *JAMA Netw Open*. 2022;5(3):e224584. doi:10.1001/jamanetworkopen.2022.4584

eMethods. Exclusion Criteria

eTable. Grouping of *International Classification of Disease, Version 10* Codes Used to Exclude Patients With Ductal-Dependent Cardiac Lesions From Analysis

eAppendix. Stata Log Files

This supplementary material has been provided by the authors to give readers additional information about their work.

© 2022 Andrist E et al. *JAMA Network Open*.

eMethods

Exclusion Criteria

Neonatal intensive care unit patients and patients with ductal-dependent congenital cardiac disease were excluded. The most common types of ductal-dependent congenital cardiac disease were excluded based on the presence of International Classification of Disease version 10 diagnosis codes associated with the hospitalization (**eTable 1**).

Supplementary Methods Information

Within our system, SpO_2 readings are automatically transferred to the electronic health record, and measurements are manually validated. We performed analyses at the individual SpO_2 reading level and at the patient level. When comparing rates of occult hypoxemia at the SpO_2 reading level, the single closest SpO_2 value was paired to an arterial blood gas (ABG) performed within 10 minutes. Multiple SpO_2 and SaO_2 pairs were available for some patients if several ABGs were performed. In the SpO_2 reading level analysis, we accounted for clustering of pairs within patients by using mixed effects models, adjusting for age, sex, SpO_2 value, and Vasoactive Infusion Score (VIS). When comparing rates of occult hypoxemia at the patient level, we adjusted for age, sex, mean SpO_2 of the patient, mean VIS of the patient, and the number of pairs available for the patient.

© 2022 Andrist E et al. *JAMA Network Open*.

eTable. Grouping of International Classification of Disease, version 10 codes used to exclude patients with ductal-dependent cardiac lesions from analysis.

Diagnosis Code	Diagnostic Category	Excluded Anomalies
P29.30	Persistent pulmonary hypertension of the newborn	
Q20*	Congenital malformations of the cardiac chambers and connections	Truncus arteriosus<br>Double outlet right ventricle<br>Transposition of the great arteries
Q21.1	Common atrioventricular canal	
Q21.3	Tetralogy of Fallot	
Q22*	Congenital malformations of the pulmonary and tricuspid valves	Tricuspid atresia<br>Ebstein’s anomaly
Q23*	Congenital malformations of aortic and mitral valves	Hypoplastic left heart syndrome
Q25.2*	Atresia of aorta	Interrupted aortic arch
Q26.2	Total anomalous pulmonary venous return	

© 2022 Andrist E et al. *JAMA Network Open*.

eAppendix. STATA log file

```
---------------------------------------------------------------------------
-----------------------------------------
      name:  <unnamed>
       log:  C:\Users\msjoding\Dropbox\active-
projects\spo2\peds\logs\final-analysis-peds-revision-copyediting.log
  log type:  text
 opened on:  21 Feb 2022, 13:10:11

. ***Final analysis for the pediatric pulse oximetry revision
.
.
. ***First reproduce all the results reported in the Main Text
. use "Z:\project-datasets\peds-pulse-cookiecutter\data\stata\final-
analytic-file-revision.dta", clear

. ***total ABG/SpO2 pairs
. count
  9,023

. unique patientid
Number of unique values of patientid is  1061
Number of records is  9023

. ***Unique black and white patients, and patient characteristics
. unique patientid if black==1
Number of unique values of patientid is  183
Number of records is  2005

. unique patientid if black==0
Number of unique values of patientid is  878
Number of records is  7018

.
. tab black if pt_tag==1

      black |      Freq.     Percent        Cum.
------------+-----------------------------------
          0 |        878       82.75       82.75
          1 |        183       17.25      100.00
------------+-----------------------------------
      Total |      1,061      100.00

. tab male if pt_tag==1

       male |      Freq.     Percent        Cum.
------------+-----------------------------------
          0 |        489       46.09       46.09
          1 |        572       53.91      100.00
------------+-----------------------------------
      Total |      1,061      100.00

.
```

© 2022 Andrist E et al. *JAMA Network Open*.

```
. sum age if pt_tag==1

    Variable |        Obs        Mean    Std. Dev.       Min        Max
-------------+---------------------------------------------------------
         age |      1,061    6.983614    6.084814    .000912   17.99384

.
. ***Time between SpO2 and ABG
. sum time_diff if black==0, d

                          time_diff
-------------------------------------------------------------
      Percentiles      Smallest
 1%            0              0
 5%            0              0
10%            0              0       Obs               7,018
25%            2              0       Sum of Wgt.       7,018

50%            4                      Mean           4.077942
                        Largest       Std. Dev.      2.848919
75%            6              9
90%            8              9       Variance       8.116341
95%            9              9       Skewness       .1646849
99%            9              9       Kurtosis       1.829172

. sum time_diff if black==1, d

                          time_diff
-------------------------------------------------------------
      Percentiles      Smallest
 1%            0              0
 5%            0              0
10%            0              0       Obs               2,005
25%            1              0       Sum of Wgt.       2,005

50%            4                      Mean           3.968579
                        Largest       Std. Dev.      2.888222
75%            6              9
90%            8              9       Variance       8.341827
95%            9              9       Skewness       .2302213
99%            9              9       Kurtosis       1.842725

.
. ***Overall mean bias and statistical significance (clustering by
patient)
. tab black, sum(bias)

            |          Summary of bias
      black |        Mean   Std. Dev.       Freq.
------------+------------------------------------
          0 |   3.4659447   5.0175866       7,018
          1 |   4.3039401   5.0371751       2,005
------------+------------------------------------
      Total |   3.6521556   5.0337378       9,023
```

© 2022 Andrist E et al. *JAMA Network Open*.

```
. mixed bias i.black || patientid:

Performing EM optimization:

Performing gradient-based optimization:

Iteration 0:   log likelihood = -26824.308
Iteration 1:   log likelihood = -26824.307

Computing standard errors:

Mixed-effects ML regression                     Number of obs     =
9,023
Group variable: patientid                       Number of groups  =
1,061

                                                Obs per group:
                                                              min =
1
                                                              avg =
8.5
                                                              max =
157

                                                Wald chi2(1)      =
13.45
Log likelihood = -26824.307                     Prob > chi2       =
0.0002

------------------------------------------------------------------------------
----
        bias |      Coef.   Std. Err.      z    P>|z|     [95% Conf.
Interval]
-------------+----------------------------------------------------------------
----
     1.black |   .8758745   .2387963     3.67   0.000     .4078422
1.343907
       _cons |    3.07794   .1017378    30.25   0.000     2.878538
3.277343
------------------------------------------------------------------------------
----

------------------------------------------------------------------------------
----
  Random-effects Parameters  |   Estimate   Std. Err.     [95% Conf.
Interval]
-----------------------------+------------------------------------------------
----
patientid: Identity          |
                  var(_cons) |   3.823654    .319692      3.245716
4.504501
-----------------------------+------------------------------------------------
----
```

© 2022 Andrist E et al. *JAMA Network Open*.

```
              var(Residual) |   20.63521   .3199606      20.01753
21.27195
--------------------------------------------------------------------------
----
LR test vs. linear model: chibar2(01) = 1078.50       Prob >= chibar2 =
0.0000

. ***p-value is for 1.black and is <0.000
.
. ***Frequency of occult hypoxemia (SaO2 < 88 & SpO2 > 91) at the SpO2
measurement level
. gen occult_hypoxemia = 1 if abg_sat < 88 & spo2>91
(8,512 missing values generated)

. replace occult_hypoxemia = 0 if abg_sat >= 88 & spo2>91
(7,198 real changes made)

.
. ***frequency of occult hypoxemia with confidence intervals at the
. proportion occult_hypoxemia if black==0, vce(cluster patientid)

Proportion estimation                 Number of obs   =      6,058

                (Std. Err. adjusted for 860 clusters in patientid)
------------------------------------------------------------------
                 |              Robust             Logit
                 | Proportion   Std. Err.     [95% Conf. Interval]
-----------------+------------------------------------------------
occult_hypoxemia |
              0  |    .941895   .0068366      .9269302    .9539473
              1  |    .058105   .0068366      .0460527    .0730698
------------------------------------------------------------------

. proportion occult_hypoxemia if black==1, vce(cluster patientid)

Proportion estimation                 Number of obs   =      1,651

                (Std. Err. adjusted for 180 clusters in patientid)
------------------------------------------------------------------
                 |              Robust             Logit
                 | Proportion   Std. Err.     [95% Conf. Interval]
-----------------+------------------------------------------------
occult_hypoxemia |
              0  |   .9036947    .020537      .8548699    .9372986
              1  |   .0963053    .020537      .0627014    .1451301
------------------------------------------------------------------

.
. ***adjusted odds ratio for occult hypoxemia at the SpO2 measurement
level
. melogit occult_hypoxemia i.black age male spo2 vaso_score || patientid:,
or

Fitting fixed-effects model:
```

© 2022 Andrist E et al. *JAMA Network Open*.

```
Iteration 0:   log likelihood = -1763.6627
Iteration 1:   log likelihood = -1485.7944
Iteration 2:   log likelihood = -1482.9467
Iteration 3:   log likelihood = -1482.9433
Iteration 4:   log likelihood = -1482.9433

Refining starting values:

Grid node 0:   log likelihood = -1374.5974

Fitting full model:

Iteration 0:   log likelihood = -1374.5974
Iteration 1:   log likelihood =  -1342.648
Iteration 2:   log likelihood = -1339.6531
Iteration 3:   log likelihood = -1339.5923
Iteration 4:   log likelihood = -1339.5925

Mixed-effects logistic regression               Number of obs     =
7,709
Group variable:       patientid                 Number of groups  =
1,040

                                                Obs per group:
                                                              min =
1
                                                              avg =
7.4
                                                              max =
124

Integration method: mvaghermite                 Integration pts.  =
7

                                                Wald chi2(5)      =
416.33
Log likelihood = -1339.5925                     Prob > chi2       =
0.0000
----------------------------------------------------------------------------
--------
occult_hypoxemia | Odds Ratio   Std. Err.      z    P>|z|     [95% Conf.
Interval]
-----------------+----------------------------------------------------------
--------
         1.black |   2.161581   .5130718     3.25   0.001     1.357472
3.442011
             age |   .9027168   .0156713    -5.90   0.000     .8725183
.9339605
            male |     .90669   .1756732    -0.51   0.613      .620207
1.325504
            spo2 |   .6139003   .0154504   -19.39   0.000     .5843529
.6449418
```

© 2022 Andrist E et al. *JAMA Network Open*.

```
      vaso_score |   1.018303   .0041236     4.48   0.000     1.010252
1.026417
           _cons |   1.11e+19   2.68e+19    18.20   0.000     9.89e+16
1.25e+21
-----------------+----------------------------------------------------------------
--------
patientid        |
       var(_cons)|   1.910633   .3311328                      1.360364
2.683487
----------------------------------------------------------------------------------
--------
Note: Estimates are transformed only in the first equation.
Note: _cons estimates baseline odds (conditional on zero random effects).
LR test vs. logistic model: chibar2(01) = 286.70      Prob >= chibar2 =
0.0000

.
. ***Frequency of occult hypoxemia at the patient level
. bysort patientid: egen any_occult_hypoxemia = mean(occult_hypoxemia)
(100 missing values generated)

. replace any_occult_hypoxemia = 1 if any_occult_hypoxemia > 0 &
any_occult_hypoxemia!=.
(4,209 real changes made)

.
. tab any_occult_hypoxemia black if pt_tag==1, col

+-------------------+
| Key               |
|-------------------|
|     frequency     |
| column percentage |
+-------------------+

any_occult |         black
_hypoxemia |         0          1 |     Total
-----------+----------------------+----------
         0 |       726        142 |       868
           |     84.42      78.89 |     83.46
-----------+----------------------+----------
         1 |       134         38 |       172
           |     15.58      21.11 |     16.54
-----------+----------------------+----------
     Total |       860        180 |     1,040
           |    100.00     100.00 |    100.00

.
. ***frequency of occult hypoxemia with confidence intervals at the
patient level
. proportion any_occult_hypoxemia if black==0 & pt_tag==1

Proportion estimation                     Number of obs   =        860
```

© 2022 Andrist E et al. *JAMA Network Open*.

```
-----------------------------------------------------------------------
                     |                                     Logit
                     | Proportion   Std. Err.     [95% Conf. Interval]
---------------------+-------------------------------------------------
any_occult_hypoxemia |
                  0  |    .844186   .0123672      .8183444    .8669492
                  1  |    .155814   .0123672      .1330508    .1816556
-----------------------------------------------------------------------

. proportion any_occult_hypoxemia if black==1 & pt_tag==1

Proportion estimation                     Number of obs   =        180

-----------------------------------------------------------------------
                     |                                     Logit
                     | Proportion   Std. Err.     [95% Conf. Interval]
---------------------+-------------------------------------------------
any_occult_hypoxemia |
                  0  |   .7888889   .0304178      .7226877    .8427256
                  1  |   .2111111   .0304178      .1572744    .2773123
-----------------------------------------------------------------------


.
. ***Adjusted odds of occult hypoxemia at the patient level
. bysort patientid: egen mean_spo2 = mean(spo2)

. bysort patientid: egen mean_vaso = mean(vaso_score)

.
. logit any_occult i.black age male total_pairs mean_spo2 mean_vaso if
pt_tag==1, or

Iteration 0:   log likelihood = -466.43157
Iteration 1:   log likelihood = -318.38666
Iteration 2:   log likelihood = -308.67026
Iteration 3:   log likelihood = -308.55943
Iteration 4:   log likelihood = -308.55931
Iteration 5:   log likelihood = -308.55931

Logistic regression                                 Number of obs     =
1,040
                                                    LR chi2(6)        =
315.74
                                                    Prob > chi2       =
0.0000
Log likelihood = -308.55931                         Pseudo R2         =
0.3385

---------------------------------------------------------------------------
------------
any_occult_hypoxemia | Odds Ratio   Std. Err.      z    P>|z|     [95%
Conf. Interval]
---------------------+-----------------------------------------------------
------------
```

© 2022 Andrist E et al. *JAMA Network Open*.

```
             1.black |   1.792397   .4752698     2.20   0.028     1.065935
3.013962
                 age |   .9301472   .0179231    -3.76   0.000     .8956737
.9659476
                male |   1.097764   .2330748     0.44   0.660     .7240757
1.66431
         total_pairs |   1.078064   .0093047     8.71   0.000     1.059981
1.096456
           mean_spo2 |   .7239936   .0286841    -8.15   0.000     .6699012
.7824538
           mean_vaso |   1.015047   .0089184     1.70   0.089     .9977169
1.032678
               _cons |   3.82e+12   1.45e+13     7.61   0.000     2.19e+09
6.66e+15
---------------------------------------------------------------------------
------------
Note: _cons estimates baseline odds.

.
.
. ***Results in the Table
.
. ***Unique hospitalizations
. unique encounterid if black==0
Number of unique values of encounterid is  934
Number of records is  7018

. unique encounterid if black==1
Number of unique values of encounterid is  186
Number of records is  2005


.
. ***Unique patients
. unique patientid if black==0
Number of unique values of patientid is  878
Number of records is  7018

. unique patientid if black==1
Number of unique values of patientid is  183
Number of records is  2005


.
. ***Sex
. tab sex black if hosp_tag==1, col chi

+-------------------+
| Key               |
|-------------------|
|     frequency     |
| column percentage |
+-------------------+

           |         black
       sex |         0          1 |     Total
```

© 2022 Andrist E et al. *JAMA Network Open*.

```
-----------+----------------------+----------
    Female |       426         90 |       516 
           |     45.61      48.39 |     46.07 
-----------+----------------------+----------
      Male |       508         96 |       604 
           |     54.39      51.61 |     53.93 
-----------+----------------------+----------
     Total |       934        186 |     1,120 
           |    100.00     100.00 |    100.00 

          Pearson chi2(1) =   0.4814   Pr = 0.488

. 
. ***Age
. tab age6 black if hosp_tag==1, col chi

+-------------------+
| Key               |
|-------------------|
|     frequency     |
| column percentage |
+-------------------+

 RECODE of |         black
       age |         0          1 |     Total
-----------+----------------------+----------
      <6mo |       152         33 |       185 
           |     16.27      17.74 |     16.52 
-----------+----------------------+----------
   6mo-1yr |        83         16 |        99 
           |      8.89       8.60 |      8.84 
-----------+----------------------+----------
   1yr-2yr |        75         12 |        87 
           |      8.03       6.45 |      7.77 
-----------+----------------------+----------
       2-4 |       152         29 |       181 
           |     16.27      15.59 |     16.16 
-----------+----------------------+----------
      4-11 |       207         42 |       249 
           |     22.16      22.58 |     22.23 
-----------+----------------------+----------
       12+ |       265         54 |       319 
           |     28.37      29.03 |     28.48 
-----------+----------------------+----------
     Total |       934        186 |     1,120 
           |    100.00     100.00 |    100.00 

          Pearson chi2(5) =   0.7946   Pr = 0.977

. 
. ***Do not check the maximum respiratory support or VIS here
. 
. ***SpO2 pair analysis
. tab black
```

© 2022 Andrist E et al. *JAMA Network Open*.

```
      black |      Freq.     Percent        Cum.
------------+-----------------------------------
          0 |      7,018       77.78       77.78
          1 |      2,005       22.22      100.00
------------+-----------------------------------
      Total |      9,023      100.00

.
. gen sp88 = sp4==1

. tab sp88 black, col chi

+-------------------+
| Key               |
|-------------------|
|     frequency     |
| column percentage |
+-------------------+

           |         black
      sp88 |         0          1 |     Total
-----------+----------------------+----------
         0 |     6,392      1,757 |     8,149
           |     91.08      87.63 |     90.31
-----------+----------------------+----------
         1 |       626        248 |       874
           |      8.92      12.37 |      9.69
-----------+----------------------+----------
     Total |     7,018      2,005 |     9,023
           |    100.00     100.00 |    100.00

          Pearson chi2(1) =  21.2075   Pr = 0.000

. melogit sp88 i.black || patientid:, or

Fitting fixed-effects model:

Iteration 0:   log likelihood = -2895.3115
Iteration 1:   log likelihood = -2860.4917
Iteration 2:   log likelihood = -2860.4807
Iteration 3:   log likelihood = -2860.4807

Refining starting values:

Grid node 0:   log likelihood = -2210.0679

Fitting full model:

Iteration 0:   log likelihood = -2210.0679
Iteration 1:   log likelihood = -2047.3725
Iteration 2:   log likelihood = -2011.5891
Iteration 3:   log likelihood = -2004.0467
Iteration 4:   log likelihood = -2004.0014
```

© 2022 Andrist E et al. *JAMA Network Open*.

```
Iteration 5:   log likelihood = -2004.0043
Iteration 6:   log likelihood = -2004.0044

Mixed-effects logistic regression               Number of obs     =
9,023
Group variable:       patientid                 Number of groups  =
1,061

                                                Obs per group:
                                                              min =
1
                                                              avg =
8.5
                                                              max =
157

Integration method: mvaghermite                 Integration pts.  =
7

                                                Wald chi2(1)      =
0.14
Log likelihood = -2004.0044                     Prob > chi2       =
0.7122
---------------------------------------------------------------------------
----
        sp88 | Odds Ratio   Std. Err.      z    P>|z|     [95% Conf.
Interval]
-------------+-------------------------------------------------------------
----
     1.black |   .8868735   .2886444    -0.37   0.712     .4686269
1.678403
       _cons |   .0101667   .0021728   -21.47   0.000     .0066875
.0154559
-------------+-------------------------------------------------------------
----
patientid    |
   var(_cons)|   5.798806   .8297689                       4.38064
7.676082
---------------------------------------------------------------------------
----
Note: Estimates are transformed only in the first equation.
Note: _cons estimates baseline odds (conditional on zero random effects).
LR test vs. logistic model: chibar2(01) = 1712.95     Prob >= chibar2 =
0.0000

.
. tab occult_88_91 if black==1

occult_88_9 |
          1 |      Freq.     Percent        Cum.
------------+-----------------------------------
          0 |         32       30.19       30.19
          1 |         74       69.81      100.00
------------+-----------------------------------
```

© 2022 Andrist E et al. *JAMA Network Open*.

```
      Total |        106      100.00

. tab occult_88_91 if black==0

occult_88_9 |
          1 |      Freq.     Percent        Cum.
------------+-----------------------------------
          0 |        146       43.71       43.71
          1 |        188       56.29      100.00
------------+-----------------------------------
      Total |        334      100.00

. tab occult_88_91

occult_88_9 |
          1 |      Freq.     Percent        Cum.
------------+-----------------------------------
          0 |        178       40.45       40.45
          1 |        262       59.55      100.00
------------+-----------------------------------
      Total |        440      100.00

.
. tab occult_92_96 if black==1

occult_92_9 |
          6 |      Freq.     Percent        Cum.
------------+-----------------------------------
          0 |        306       70.67       70.67
          1 |        127       29.33      100.00
------------+-----------------------------------
      Total |        433      100.00

. tab occult_92_96 if black==0

occult_92_9 |
          6 |      Freq.     Percent        Cum.
------------+-----------------------------------
          0 |      1,597       86.56       86.56
          1 |        248       13.44      100.00
------------+-----------------------------------
      Total |      1,845      100.00

. tab occult_92_96

occult_92_9 |
          6 |      Freq.     Percent        Cum.
------------+-----------------------------------
          0 |      1,903       83.54       83.54
          1 |        375       16.46      100.00
------------+-----------------------------------
      Total |      2,278      100.00

.
```

© 2022 Andrist E et al. *JAMA Network Open*.

```
. tab occult_97_100 if black==1

occult_97_1 |
         00 |      Freq.     Percent        Cum.
------------+-----------------------------------
          0 |      1,186       97.37       97.37
          1 |         32        2.63      100.00
------------+-----------------------------------
      Total |      1,218      100.00

. tab occult_97_100 if black==0

occult_97_1 |
         00 |      Freq.     Percent        Cum.
------------+-----------------------------------
          0 |      4,109       97.53       97.53
          1 |        104        2.47      100.00
------------+-----------------------------------
      Total |      4,213      100.00

. tab occult_97_100

occult_97_1 |
         00 |      Freq.     Percent        Cum.
------------+-----------------------------------
          0 |      5,295       97.50       97.50
          1 |        136        2.50      100.00
------------+-----------------------------------
      Total |      5,431      100.00

.
. tab occult_hypoxemia if black==1

occult_hypo |
      xemia |      Freq.     Percent        Cum.
------------+-----------------------------------
          0 |      1,492       90.37       90.37
          1 |        159        9.63      100.00
------------+-----------------------------------
      Total |      1,651      100.00

. tab occult_hypoxemia if black==0

occult_hypo |
      xemia |      Freq.     Percent        Cum.
------------+-----------------------------------
          0 |      5,706       94.19       94.19
          1 |        352        5.81      100.00
------------+-----------------------------------
      Total |      6,058      100.00

. tab occult_hypoxemia black, chi

occult_hyp |         black
```

© 2022 Andrist E et al. *JAMA Network Open*.

```
    oxemia |         0          1 |     Total
-----------+----------------------+----------
         0 |     5,706      1,492 |     7,198
         1 |       352        159 |       511
-----------+----------------------+----------
     Total |     6,058      1,651 |     7,709

          Pearson chi2(1) =  30.5897   Pr = 0.000

.
. melogit occult_hypoxemia black || patientid:

Fitting fixed-effects model:

Iteration 0:   log likelihood = -1953.5396
Iteration 1:   log likelihood = -1866.9971
Iteration 2:   log likelihood = -1866.3695
Iteration 3:   log likelihood = -1866.3691
Iteration 4:   log likelihood = -1866.3691

Refining starting values:

Grid node 0:   log likelihood = -1661.4725

Fitting full model:

Iteration 0:   log likelihood = -1661.4725
Iteration 1:   log likelihood = -1597.6282
Iteration 2:   log likelihood = -1588.3219
Iteration 3:   log likelihood = -1587.6292
Iteration 4:   log likelihood = -1587.6336
Iteration 5:   log likelihood = -1587.6336

Mixed-effects logistic regression               Number of obs     =
7,709
Group variable:       patientid                 Number of groups  =
1,040

                                                Obs per group:
                                                              min =
1
                                                              avg =
7.4
                                                              max =
124

Integration method: mvaghermite                 Integration pts.  =
7

                                                Wald chi2(1)      =
4.65
Log likelihood = -1587.6336                     Prob > chi2       =
0.0310
```

© 2022 Andrist E et al. *JAMA Network Open*.

```
--------------------------------------------------------------------------
--------
occult_hypoxemia |      Coef.   Std. Err.      z    P>|z|     [95% Conf.
Interval]
-----------------+--------------------------------------------------------
--------
           black |   .5603347   .2597228     2.16   0.031     .0512874
1.069382
           _cons |  -4.215714   .1820746   -23.15   0.000    -4.572574   -
3.858854
-----------------+--------------------------------------------------------
--------
patientid       |
       var(_cons)|     3.2961   .5097339                      2.434244
4.4631
--------------------------------------------------------------------------
--------
LR test vs. logistic model: chibar2(01) = 557.47      Prob >= chibar2 =
0.0000

.
. ***Spot check rate calculations in the figure
.
. ***overall
. proportion occult_hypoxemia if black==0

Proportion estimation                Number of obs   =      6,058

------------------------------------------------------------------
                 |                                  Logit
                 | Proportion   Std. Err.     [95% Conf. Interval]
-----------------+------------------------------------------------
occult_hypoxemia |
              0  |    .941895   .0030057      .9357147    .9475145
              1  |    .058105   .0030057      .0524855    .0642853
------------------------------------------------------------------

. proportion occult_hypoxemia if black==1

Proportion estimation                Number of obs   =      1,651

------------------------------------------------------------------
                 |                                  Logit
                 | Proportion   Std. Err.     [95% Conf. Interval]
-----------------+------------------------------------------------
occult_hypoxemia |
              0  |   .9036947   .0072604      .8884832    .9170251
              1  |   .0963053   .0072604      .0829749    .1115168
------------------------------------------------------------------

.
. ***@spo2==92
. proportion occult_hypoxemia if black==0 & spo2==92
```

© 2022 Andrist E et al. *JAMA Network Open*.

```
Proportion estimation               Number of obs   =        210

-----------------------------------------------------------------
                 |                                   Logit
                 | Proportion   Std. Err.     [95% Conf. Interval]
-----------------+-----------------------------------------------
occult_hypoxemia |
              0  |   .6428571    .033065      .5753897    .7050988
              1  |   .3571429    .033065      .2949012    .4246103
-----------------------------------------------------------------

. proportion occult_hypoxemia if black==1 & spo2==92

Proportion estimation               Number of obs   =         62

-----------------------------------------------------------------
                 |                                   Logit
                 | Proportion   Std. Err.     [95% Conf. Interval]
-----------------+-----------------------------------------------
occult_hypoxemia |
              0  |    .483871    .063467      .3606122    .6091238
              1  |    .516129    .063467      .3908762    .6393878
-----------------------------------------------------------------

.
. ***@spo2==93
. proportion occult_hypoxemia if black==0 & spo2==93

Proportion estimation               Number of obs   =        266

-----------------------------------------------------------------
                 |                                   Logit
                 | Proportion   Std. Err.     [95% Conf. Interval]
-----------------+-----------------------------------------------
occult_hypoxemia |
              0  |   .7518797   .0264828      .6961767    .8002996
              1  |   .2481203   .0264828      .1997004    .3038233
-----------------------------------------------------------------

. proportion occult_hypoxemia if black==1 & spo2==93

Proportion estimation               Number of obs   =         66

-----------------------------------------------------------------
                 |                                   Logit
                 | Proportion   Std. Err.     [95% Conf. Interval]
-----------------+-----------------------------------------------
occult_hypoxemia |
              0  |   .5909091   .0605199      .4669805    .7042711
              1  |   .4090909   .0605199      .2957289    .5330195
-----------------------------------------------------------------

.
. ***@spo2==94
```

© 2022 Andrist E et al. *JAMA Network Open*.

```
. proportion occult_hypoxemia if black==0 & spo2==94

Proportion estimation                Number of obs   =        326

------------------------------------------------------------------
                 |                                  Logit
                 | Proportion   Std. Err.     [95% Conf. Interval]
-----------------+------------------------------------------------
occult_hypoxemia |
              0  |   .8773006   .0181713      .8368556    .9088115
              1  |   .1226994   .0181713      .0911885    .1631444
------------------------------------------------------------------

. proportion occult_hypoxemia if black==1 & spo2==94

Proportion estimation                Number of obs   =         72

------------------------------------------------------------------
                 |                                  Logit
                 | Proportion   Std. Err.     [95% Conf. Interval]
-----------------+------------------------------------------------
occult_hypoxemia |
              0  |   .6805556   .0549494      .5627528    .7790796
              1  |   .3194444   .0549494      .2209204    .4372472
------------------------------------------------------------------

.
. ***@spo2==95
. proportion occult_hypoxemia if black==0 & spo2==95

Proportion estimation                Number of obs   =        472

------------------------------------------------------------------
                 |                                  Logit
                 | Proportion   Std. Err.     [95% Conf. Interval]
-----------------+------------------------------------------------
occult_hypoxemia |
              0  |   .9237288   .0122175      .8959831    .9445317
              1  |   .0762712   .0122175      .0554683    .1040169
------------------------------------------------------------------

. proportion occult_hypoxemia if black==1 & spo2==95

Proportion estimation                Number of obs   =        108

------------------------------------------------------------------
                 |                                  Logit
                 | Proportion   Std. Err.     [95% Conf. Interval]
-----------------+------------------------------------------------
occult_hypoxemia |
              0  |    .787037   .0393947      .6987223    .8548419
              1  |    .212963   .0393947      .1451581    .3012777
------------------------------------------------------------------
```

© 2022 Andrist E et al. *JAMA Network Open*.

```
.
. ***@spo2==96
. proportion occult_hypoxemia if black==0 & spo2==96

Proportion estimation                Number of obs   =        571

------------------------------------------------------------------
                 |                                 Logit
                 | Proportion   Std. Err.     [95% Conf. Interval]
-----------------+------------------------------------------------
occult_hypoxemia |
              0  |   .9457093   .0094825      .9237785    .9615924
              1  |   .0542907   .0094825      .0384076    .0762215
------------------------------------------------------------------

. proportion occult_hypoxemia if black==1 & spo2==96

Proportion estimation                Number of obs   =        125

------------------------------------------------------------------
                 |                                 Logit
                 | Proportion   Std. Err.     [95% Conf. Interval]
-----------------+------------------------------------------------
occult_hypoxemia |
              0  |       .824   .0340616      .7462698    .8816926
              1  |       .176   .0340616      .1183074    .2537302
------------------------------------------------------------------

.
. ***@spo2==97
. proportion occult_hypoxemia if black==0 & spo2==97

Proportion estimation                Number of obs   =        689

------------------------------------------------------------------
                 |                                 Logit
                 | Proportion   Std. Err.     [95% Conf. Interval]
-----------------+------------------------------------------------
occult_hypoxemia |
              0  |   .9622642   .0072596       .945119    .9741993
              1  |   .0377358   .0072596      .0258007     .054881
------------------------------------------------------------------

. proportion occult_hypoxemia if black==1 & spo2==97

Proportion estimation                Number of obs   =        156

------------------------------------------------------------------
                 |                                 Logit
                 | Proportion   Std. Err.     [95% Conf. Interval]
-----------------+------------------------------------------------
occult_hypoxemia |
              0  |   .9358974   .0196105      .8844549    .9653346
              1  |   .0641026   .0196105      .0346654    .1155451
```

© 2022 Andrist E et al. *JAMA Network Open*.

```
------------------------------------------------------------------

.
. ***@spo2==98
. proportion occult_hypoxemia if black==0 & spo2==98

Proportion estimation                Number of obs   =        823

------------------------------------------------------------------
                 |                                   Logit
                 | Proportion   Std. Err.     [95% Conf. Interval]
-----------------+------------------------------------------------
occult_hypoxemia |
               0 |   .9708384   .0058652      .9568378    .9803907
               1 |   .0291616   .0058652      .0196093    .0431622
------------------------------------------------------------------

. proportion occult_hypoxemia if black==1 & spo2==98

Proportion estimation                Number of obs   =        203

------------------------------------------------------------------
                 |                                   Logit
                 | Proportion   Std. Err.     [95% Conf. Interval]
-----------------+------------------------------------------------
occult_hypoxemia |
               0 |   .9704433   .0118868      .9354932     .986726
               1 |   .0295567   .0118868       .013274    .0645068
------------------------------------------------------------------

.
. ***@spo2==99
. proportion occult_hypoxemia if black==0 & spo2==99

Proportion estimation                Number of obs   =        925

------------------------------------------------------------------
                 |                                   Logit
                 | Proportion   Std. Err.     [95% Conf. Interval]
-----------------+------------------------------------------------
occult_hypoxemia |
               0 |   .9751351   .0051198      .9628423    .9834311
               1 |   .0248649   .0051198      .0165689    .0371577
------------------------------------------------------------------

. proportion occult_hypoxemia if black==1 & spo2==99

Proportion estimation                Number of obs   =        257

------------------------------------------------------------------
                 |                                   Logit
                 | Proportion   Std. Err.     [95% Conf. Interval]
-----------------+------------------------------------------------
occult_hypoxemia |
```

© 2022 Andrist E et al. *JAMA Network Open*.

```
              0  |   .9727626   .0101536      .9437941    .9870063
              1  |   .0272374   .0101536      .0129937    .0562059
------------------------------------------------------------------

.
. ***@spo2==100
. proportion occult_hypoxemia if black==0 & spo2==100

Proportion estimation                Number of obs   =      1,776

------------------------------------------------------------------
                 |                                   Logit
                 | Proportion   Std. Err.     [95% Conf. Interval]
-----------------+------------------------------------------------
occult_hypoxemia |
              0  |    .982545   .0031075      .9752809    .9877014
              1  |    .017455   .0031075      .0122986    .0247191
------------------------------------------------------------------

. proportion occult_hypoxemia if black==1 & spo2==100

Proportion estimation                Number of obs   =        602

------------------------------------------------------------------
                 |                                   Logit
                 | Proportion   Std. Err.     [95% Conf. Interval]
-----------------+------------------------------------------------
occult_hypoxemia |
              0  |   .9850498    .004946      .9714846    .9922136
              1  |   .0149502    .004946      .0077864    .0285154
------------------------------------------------------------------

.
. log close
      name:  <unnamed>
       log:  C:\Users\msjoding\Dropbox\active-
projects\spo2\peds\logs\final-analysis-peds-revision-copyediting.log
  log type:  text
 closed on:  21 Feb 2022, 13:10:18
--------------------------------------------------------------------------
-----------------------------------------
```

© 2022 Andrist E et al. *JAMA Network Open*.
